# Onabotulinumtoxin A for the management of chronic migraine in current clinical practice: results of a survey of sixty-three Italian headache centers

**DOI:** 10.1186/s10194-017-0773-7

**Published:** 2017-06-30

**Authors:** Cristina Tassorelli, Marco Aguggia, Marina De Tommaso, Pierangelo Geppetti, Licia Grazzi, Luigi Alberto Pini, Paola Sarchielli, Gioacchino Tedeschi, Paolo Martelletti, Pietro Cortelli

**Affiliations:** 1 Headache Science Center, National Neurological Institute C. Mondino, Pavia, Italy; 2 Department of Brain and Behavioral Sciences, University of Pavia, Via Mondino 2, 27100 Pavia, Italy; 3 Headache Center, Neurology Department, Asti Hospital, Asti, Italy; 4 Applied Neurophysiology and Pain Unit, SMBNOS Department, Polyclinic General Hospital, Bari Aldo Moro University, Bari, Italy; 5 Headache Center, Department of Health Sciences, University of Florence, Florence, Italy; 6 Headache and Neuroalgology Unit, Neurological Institute “C. Besta” IRCCS Foundation, Milan, Italy; 7 Center for Neuroscience and Neurotechnology, Polyclinic Hospital, University of Modena and Reggio Emilia, Modena, Italy; 8 Neurology Clinic, University Hospital of Perugia, Perugia, Italy; 9 Department of Medical, Surgical, Neurological, Metabolic and Aging Sciences, University of Campania “Luigi Vanvitelli”, Naples, Italy; 10 Department of Clinical and Molecular Medicine, Sapienza University of Rome and Regional Referral Headache Center, Sant’Andrea Hospital, Rome, Italy; 11 Department of Biomedical and Neuromotor Sciences, University of Bologna, Bologna, Italy; 12 IRCCS Institute of Neurological Sciences of Bologna, Bellaria Hospital, Bologna, Italy

**Keywords:** Botox, Chronic migraine, Headache, Migraine prophylaxis, Onabotulinumtoxin A

## Abstract

**Background:**

Chronic migraine is a complex clinical condition often undertreated. Onabotulinumtoxin A (OBT-A) was approved in Italy in 2013 for symptom relief in patients with chronic migraine who have failed, or do not tolerate, oral prophylactic treatments. However, the impact of OBT-A in clinical practice remains to be defined.

**Methods:**

To investigate the current management of chronic migraine with OBT-A in clinical practice, a web-based survey was conducted among clinicians working in third-level headache centers across Italy. A 26-item questionnaire was designed and developed by a group of 10 Italian headache specialists to address the following issues: treatment paradigm and OBT-A injection intervals, frequency of treatment and retreatment, definition of responders/non-responders, satisfaction with treatment potential impact of early treatment with OBT-A. Ninety-six headache centers were selected and contacted via e-mail. The online survey was anonymous and carried out using a secure website.

**Results:**

Overall, 64 of the 96 centers (66.7%) completed the questionnaire. Most centers (98.4%) had been using OBT-A for >1 year. OBT-A was administered according to the PREEMPT paradigm in most centers (88.9%). While during the first year of prophylaxis with OBT-A most clinicians (93.6%) repeated OBT-A treatment every 3 months, as recommended, in the following years interval duration was variable. Response to OBT-A was defined as a ≥ 50% reduction in the headache days by 58.7% of the clinicians, and as a ≥ 30% reduction by 25.4% of them. Almost 60% of the clinicians considered OBT-A as a long-lasting therapy, while for one-third of them treatment could be discontinued in patients showing a benefit for ≥6 months. According to 80% of the clinicians, early administration of OBT-A after the onset of chronic migraine was associated with better outcomes, and 47.6% felt that OBT-A should be recommended as a first-line option.

**Conclusions:**

This survey indicates that in third-level headache centers in Italy OBT-A is used in good compliance with current recommendations. There is agreement about the definition of response as a reduction in headache days by 30% to 50%. Additional effort is required to define response to OBT-A and to establish optimal treatment duration.

## Background

Migraine is a neurologic disorder characterized by attacks of severe pulsating unilateral headache [1]. Episodic migraine can progress to chronic migraine (CM), which is defined as headache on ≥15 days/month for ≥3 months of which ≥8 days meet criteria for migraine or respond to migraine-specific treatments [1]. CM is estimated to affect 0.9% to 2.2% of the general population and its burden on affected individuals is considerable [2–5]. The management of patients with CM poses a major challenge to headache specialists because of the complex comorbidities frequently associated with this condition, including drug overuse, anxiety, and depressive disorders [6, 7]. Furthermore, patients with CM are often poor responders to prophylactic treatments [2, 8–10].

Few evidence-based treatment options are available for CM [11–13]. In recent years, the efficacy of Onabotulinumtoxin A (OBT-A) for the prophylaxis of CM has been demonstrated in two well-designed phase III clinical trials, the Phase III Research Evaluating Migraine Prophylaxis Therapy-1 (PREEMPT) 1 and 2 trials [14, 15]. A pooled analysis of these trials showed that OBT-A was significantly more effective than placebo in reducing the mean frequency of days with headache and headache episodes [16]. The PREEMPT clinical program also established the standardized paradigm for OBT-A administration for CM prophylaxis that is currently recommended for the first 56 weeks of treatment [14, 15]. This paradigm involves the intramuscular injection of a dose of 155 U of OBT-A, administered to 31 injection sites across 7 head and neck muscles (5 U in 0.1 mL for each injection). The addition of up to 40 U OBT-A, administered to 8 additional injection sites across 3 head and neck muscles, is allowed using a follow-the-pain approach. Based on the evidence from the PREEMPT program, OBT-A was granted authorization for the prophylaxis of CM by the US Food and Drug Administration (FDA) and the European Medicines Agency (EMA) in 2010. The use of OBT-A for the prophylaxis of CM has also been endorsed by several international societies, usually as second-line treatment [17–21].

The performance of a drug, as well as the possible issues associated with its use, may vary considerably from randomized clinical trials (RCTs) to the real-world setting [22–24]. In the strictly controlled environment of RCTs, the characteristics and comorbidities of the study population, the setting, and the conditions under which a given drug is administered may differ from those typically encountered in clinical practice. Due to the relatively recent approval of OBT-A for CM, real-world data, essential for establishing the effectiveness of a novel treatment, are just beginning to emerge [25–28]. The evidence so far available supports the results from the PREEMPT clinical program [25, 29]. In Italy, OBT-A was approved in 2013 for symptom relief in patients with CM who have failed, or do not tolerate, oral prophylactic treatments [30]. Since then, several Italian headache centers have adopted OBT-A for the routine management of CM and some of them have confirmed the efficacy of this new approach as assessed by conventional outcome measures, including headache frequency, pain intensity, use of medications for symptom relief, headache-related disability, and health-related quality of life (HR-QoL) [26, 31–35]. However, data are lacking concerning the practical challenges that clinicians may have to face when using OBT-A in real life, or concerning the effectiveness of this treatment based on their judgment as well as on patient reports.

In this context, we set out to conduct a web-based survey to describe the current management of CM with OBT-A in the everyday practice of headache centers in Italy. Surveys have long been recognized as a useful tool in clinical research [36, 37]. Electronic surveys have several advantages over conventional ones (e.g., postal and telephone surveys), including the practical convenience associated with the direct storage of responses in a database, the possibility of easily contacting large samples of participants, and the rapidity of data collection [36, 37]. The specific objectives of our survey were to investigate the opinion of clinicians about the efficacy of the treatment of migraine with OBT-A, to identify unmet needs from the clinician and/or patient perspective, and to collect other practice-related suggestions to be used for further optimizing the prophylaxis of CM with OBT-A. This article presents the results of our survey.

## Methods

This was an independent survey investigating the current management of migraine treatment with OBT-A. The survey was designed and developed over the course of 2016, during two meetings organized and attended by a group of 10 Italian experts in the treatment of CM, under the auspices of the board of the *Associazione Italiana per la Ricerca sulle Cefalee* (ANIRCEF) and the *Società Italiana per lo Studio delle Cefalee* (SISC). The first meeting was virtual and took place via a web-conference, the second was a face-to-face meeting. The main objectives of the meetings were to develop a questionnaire addressing practical and controversial issues of CM treatment with OBT-A to be used for a survey, to decide, based on consensus, the methodology of the survey, and to identify the characteristics of headache centers to be invited to participate in the survey.

The questionnaire was developed based on the experience of the 10 headache experts in the management of CM in clinical practice, and on the review and discussion of the available scientific evidence concerning OBT-A for CM. During the first meeting, the following issues were discussed and identified as relevant for the current management of CM: treatment paradigm and OBT-A injection intervals, frequency of treatment and retreatment, definition of responders/non-responders, satisfaction with treatment, potential impact of early treatment with OBT-A, and availability of resources. Such issues were addressed in the questionnaire, which consisted of 26 questions (available in Additional file 1). A set of questions concerned the characteristics of the responding centers (number of patients treated, qualifications, number of years of experience). For most items, we adopted close-ended, single-choice questions. Two questions (8 and 9) allowed multiple answers and the participants were asked to rank their answers according to importance. Two other questions (23 and 24) were about the degree of satisfaction with OBT-A treatment assessed on a scale from 0 (no satisfaction) to 10 (highest satisfaction). Contents and questions of the survey were developed following published recommendations for the design and set-up of web-based surveys [38–40].

The experts agreed on the use of a web-based methodology that invited participants via an e-mail message containing a personalized link for accessing the online questionnaire. The centers to be invited to participate in the survey were identified from the registries of the two Italian scientific headache societies (ANIRCEF and SISC) [41, 42]. The following requirements had to be satisfied by the centers in order to be eligible for the survey: i) qualification as third-level headache centers (i.e., hospital-based centers in which advanced multidisciplinary care is delivered by headache specialists) [43]; ii) certification of training in the use of OBT-A in CM according to the PREEMPT paradigm obtained after attendance of the specialized courses of Continuing Medical Education delivered in Italy; iii) at least one year of experience in the use of OBT-A for the treatment of CM; and iv) routine use of a headache diary to monitor patients’ symptoms [20, 44]. Care was taken to ensure that the participating centers were located over the entire national territory so as to be representative of clinical practice across the various regions of Italy. Overall, 96 centers using OBT-A for CM and fulfilling the inclusion requirements were identified.

In April 2016, a cover letter was sent by e-mail to the chairs of the selected centers explaining the rationale and objective of the survey, and providing a personalized link to access the questionnaire and instructions on how to complete it. The questionnaire was accessible online for a period of 3 weeks. After this period, a reminder was sent by e-mail to those clinicians who had not yet responded to the survey, allowing them an additional week to complete the questionnaire. The online survey was carried out using a secure survey website (www.surveymonkey.com). In order to avoid duplication of data, we adopted the Verisign certificate version 3 and 128-bit encryption, which strictly associated a single link to a single questionnaire. The questionnaires were rigorously anonymous and did not foresee the collection of sensitive data, including identifiers of the respondents, demographic data, identifiable patient information, and geographic location. In addition, to further ensure privacy protection, questionnaires were dissociated from the original link before being processed for data analysis.

With regard to ethical issues, given the independent nature of the survey, the measures taken to ensure anonymity, and the absence of sensitive patients data in the questionnaire, and in accordance with Italian regulations, no formal approval of the survey was required from the Institutional Review Boards of the participating centers.

The results of the survey were analyzed by descriptive statistics, summarized in tables and figures as percentages. During a face-to-face meeting held in Rome in July 2016, the expert panel discussed the results of the survey.

## Results

### Response rate and characteristics of the participating centers

Of the 96 selected centers, 46 (47.9%) responded to the survey after 3 weeks. Sending a reminder improved the response rate to 66.7%, with a total of 64 out of 96 centers completing the questionnaire. All the questionnaires received were completely filled, but one center reported less than 1 year of experience with OBT-A. Answers from this center were therefore excluded from further analysis.

Most centers (61.9%) were treating moderate numbers of patients (5-20 per month) (Table 1). All centres included in the analysis had been using OBT-A for the prophylaxis of CM for more than 1 year, and 38.1% for more than 3 years. More than 80% of the centers had a dedicated facility for the administration of OBT-A to patients with CM and the majority of them (71.4%) used an electronic data recording system in their everyday practice.

**Table 1:** Characteristics of the centers that participated in the survey

Answer options	n (%)
Number of patients treated on a monthly basis with Onabotulinumtoxin A (OBT-A)	
< 5 patients	15 (23.8)
5−10 patients	21 (33.3)
10−20 patients	18 (28.6)
20−40 patients	5 (7.9)
> 40 patients	4 (6.4)
Years of experience with OBT-A for chronic migraine	
> 1 year and ≤3 years	39 (61.9)
> 3 years	24 (38.1)
Longest follow-up of patients treated with OBT-A for chronic migraine	
1 year	13 (20.6)
2 years	24 (38.1)
3 years	18 (28.6)
≥ 4 years	8 (12.7)
Availability of electronic data recording system	
Yes	45 (71.4)
No	18 (28.6)
Availability of a facility dedicated to the treatment of chronic migraine with OBT-A	
Yes	51 (81.9)
No	12 (19.1)

### Treatment characteristics

**Fig. 1:**
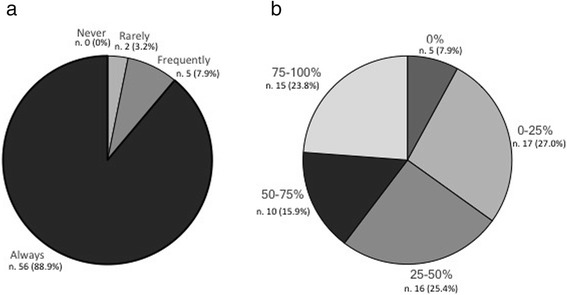
Compliance of clinicians with the PREEMPT paradigm for Onabotulinumtoxin A (OBT-A) administration: **a** frequency of use of PREEMPT paradigm; **b** proportion of patients treated with the follow-the-pain paradigm

**Fig. 2:**
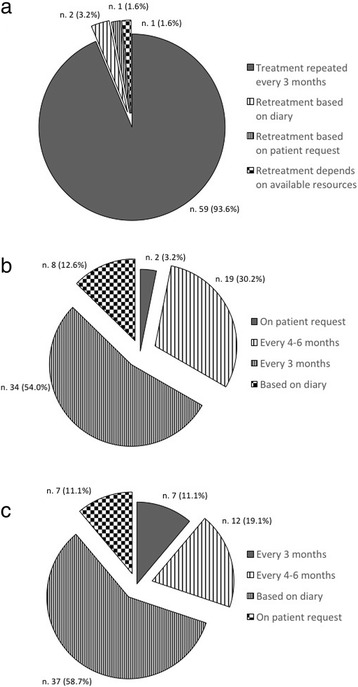
Frequency of treatment cycles during the first (**a**), second (**b**), and third (**c**) year from the beginning of prophylaxis with Onabotulinumtoxin A (OBT-A)

Most centers (88.9%) administered OBT-A exclusively according to the PREEMPT paradigm (Fig. 1a). The PREEMPT paradigm was followed frequently by 7.9% of the centers, while only 3.2% used it rarely. The follow-the-pain approach was used very frequently, with only a minority of centers never resorting to it (Fig. 1b). The interval of 3 months between treatment cycles was adopted by the large majority of the centers (93.6%) during the first year (Fig. 2a). The proportion of centers that adopted the 3-month interval decreased to 54.0% during the second year of treatment (Fig. 2b). During the third year, the centers mostly relied on the information contained in the patient clinical diary to make decisions concerning the timing of retreatment (58.7%) and, with the exception of 11.1% of them, they no longer adhered strictly to the 3-month interval between cycles (Fig. 2c).

### Indicators of treatment efficacy

**Fig. 3:**
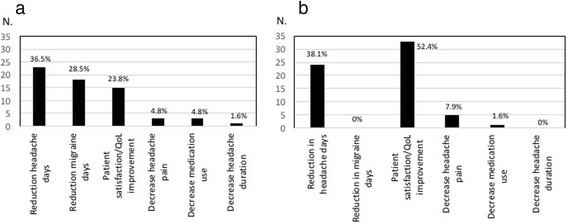
Most important indicator of treatment efficacy (**a**) for clinicians and (**b**) for patients (as assessed by clinicians)

According to the participants in the survey, the most relevant indicator of efficacy of the prophylaxis with OBT-A was the reduction in the number of days with headache, followed by the reduction in the number of days with migraine and by patient satisfaction/improvement in QoL (Fig. 3a). The clinicians completing the questionnaire had the perception, based on the feedback and comments received from their patients, that patients evaluated treatment efficacy slightly differently, with satisfaction/improvement in QoL being the most important outcome, followed by the reduction in headache days and the decrease in headache pain intensity (Fig. 3b).

### Definition of response to OBT-A

Data concerning the definition of response to OBT-A and related issues are summarized in Table 2. For nearly 60% of the participants in the survey, response to OBT-A corresponded to a ≥ 50% reduction in the number of days with headache. Notably, 1 out of 4 clinicians also considered patients who experienced a reduction in the number of headache days ≥30% as responders. Furthermore, a small proportion of clinicians also felt that a reduction <30% in headache days could be clinically relevant if associated with at least another subjective or objective indicator of improvement. According to 55.6% of the clinicians, patients should be classified as non-responders after the failure of at least 3 cycles of treatment, while about 40% of the clinicians felt that the failure of 4 or more treatment sessions is needed before classifying a patient as a non-responder. More than half of the participants (58.7%) considered the treatment with OBT-A as a therapy that should be maintained in the long-term in responders, while about one-third thought that the treatment could be discontinued in those patients showing a benefit persisting for at least 6 months (Table 2).

**Table 2:** Definition of response to treatment with Onabotulinumtoxin A (OBT-A) and treatment duration in chronic migraine

Answer options	n (%)
Reduction in the number of headache days required to define response to OBT-A	
≥ 30%	16 (25.4)
≥ 50%	37 (58.7)
< 30% provided that at least one of the following improves:	10 (15.9)
• patient satisfaction with treatment and QoL	
• intensity of headache pain	
• use of medications for symptom relief	
• duration of headache attacks	
Number of treatment cycles administered before considering a patient as a non-responder and discontinuing OBT-A	
2	1 (1.6)
3	35 (55.6)
4	14 (22.2)
> 4	13 (20.6)
Criteria adopted for discontinuing OBT-A in responders	
None, as treatment should be maintained in the long-term	37 (58.7)
Benefits for ≥6 months	21 (33.3)
After 5 treatment cycles	3 (4.8)
Achievement of <15 days/month with headache	2 (3.2)

*QoL* quality of life

### OBT-A and other prophylactic treatments

OBT-A treatment was offered by 60.3% of the clinicians after the failure of more than 3 oral prophylactic agents (Table 3). However, in most centers (71.4%), OBT-A was frequently associated with one or more additional prophylactic treatments (Table 3). Most clinicians (69.8%) considered OBT-A to be more favorable than the available oral prophylactic therapies because of a better safety profile. In addition, 80% frequently had the perception that the efficacy of OBT-A was greater when the treatment was administered earlier after the onset of CM (Table 3). Thus, almost half of the participants (47.6%) felt that OBT-A should be recommended as a first-line option for the treatment of CM.

**Table 3:** Onabotulinumtoxin A (OBT-A) in the context of other prophylactic therapies for chronic migraine and timing of OBT-A initiation

Answer options	n (%)
Frequency of combination of OBT-A with other prophylactic therapies	
Never	3 (4.8)
Rarely	14 (22.2)
Frequently	45 (71.4)
Always	1 (1.6)
Number of prophylactic therapies used before initiating OBT-A	
0	1 (1.6)
1	2 (3.2)
2-3	22 (34.9)
> 3	38 (60.3)
Rating of tolerability profile of OBT-A vs. oral prophylactic therapies	
More favorable	57 (90.5)
Comparable	5 (7.9)
Less favorable	1 (1.6)
Rating of efficacy/safety ratio of OBT-A vs. oral prophylactic therapies	
More favorable	44 (69.8)
Comparable	18 (28.6)
Less favorable	1 (1.6)
Impression of greater efficacy of OBT-A when initiated early in the course of chronic migraine	
Never	0
Rarely	9 (14.3)
Frequently	51 (80.9)
Always	3 (4.8)
Recommendation of OBT-A as first-line treatment based on the pharmacological profile	
Yes	30 (47.6)
No	33 (52.4)

### Satisfaction with OBT-A treatment

**Fig. 4:**
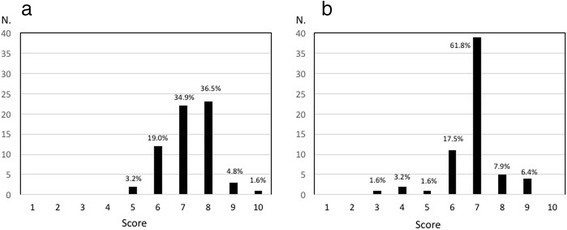
Satisfaction with Onabotulinumtoxin A (OBT-A) treatment: **a** treatment satisfaction of clinicians; **b** treatment satisfaction of patients (as perceived by clinicians). Treatment satisfaction was rated on a scale from 0 (no satisfaction) to 10 (highest satisfaction)

Satisfaction with treatment was high among clinicians, as suggested by the great proportion of participants (77.8%) with a satisfaction score ≥ 7 on a scale from 0 (totally unsatisfied) to 10 (maximum satisfaction) (Fig. 4a). Satisfaction with OBT-A treatment appeared to be high also among patients, according to the clinicians’ perception (Fig. 4b). Indeed, 76.2% of the clinicians estimated that their patients would rate treatment satisfaction with a score ≥ 7.

### Impact of resources on treatment

Overall, the centers participating in the survey reported sufficient resources to meet current and future demand (Table 4). In those centers with insufficient resources, the impact on patients was variable and ranging from longer waiting times for the first visit or follow-up visits to difficulties in respecting the exact schedule of drug administration leading, in a few cases, to the referral of patients to other centers for treatment.

**Table 4:** Resources for patient management

Answer options	Percent
Evaluation of adequacy of available resources (equipment and personnel) at center	
Able to meet current and expected future demand	33 (52.4)
Able to meet current demand, but not expected future demand	23 (36.5)
Unable to meet current and future demand	5 (7.9)
Other	2 (3.2)
Impact of inadequate resources on patient management	
Scheduled visits are postponed or cancelled	2 (3.2)
Treatment not administered according to recommended schedule	8 (12.7)
Patients not monitored with recommended timing	13 (20.6)
Long waiting times for first visit	24 (38.1)
Long waiting times for follow-up visits	10 (15.9)
Referral of new patients to other centers to avoid increase in waiting times	2 (3.2)
Other	4 (6.4)

## Discussion

CM is a highly disabling condition that affects as much as 2% of the general population [3, 5], responds poorly to prophylactic therapies [8–10], and is associated with significant costs for affected individuals and society [2, 4, 45]. A better knowledge of the use of OBT-A for migraine prophylaxis in real-life practice is of paramount importance to further optimize the effectiveness of this treatment. This survey investigated the experience acquired by third-level headache centers in Italy in the use of OBT-A since its approval.

To our knowledge, this is the first survey in the field of CM to be conducted among expert clinicians. The survey reveals the very good compliance of Italian headache centers with current recommendations on the use of OBT-A for the prophylaxis of CM, as shown by the fact that 89% of the centers participating in the survey always used the recommended PREEMPT paradigm for OBT-A administration, and 94% repeated the treatment at 3-month intervals during the first year of prophylaxis. Our findings also show that clinicians were generally satisfied with OBT-A treatment and that, in their opinion, patients as well were satisfied with it.

The survey suggests a difference between clinicians and patients in the relevance given to the various measures of efficacy. For clinicians, the most important indicator of efficacy was represented by the reduction in the number of days with headache, in agreement with the efficacy measures used in the clinical trials and in real-life studies evaluating OBT-A in CM [14, 15, 28, 31, 34, 35]. In the case of patients, the impression of clinicians was that they valued OBT-A efficacy mostly in terms of changes in QoL, a secondary outcome measure in the PREEMPT program [14, 15] which has not been extensively investigated in real-life studies. Of note, a *post-hoc* analysis of the PREEMPT studies showed a significantly positive impact of OBT-A treatment on patient health-related QoL [46].

The present survey also suggests that a number of issues related to the use of OBT-A remain unsolved in clinical practice. An important unsettled issue is how long treatment should be continued after the first year of prophylaxis with OBT-A. The survey shows that, after the first year, treatment schedules became variable with time intervals between treatment cycles ranging from 3 to 6 months. This behavior indicates a tendency of clinicians to explore whether OBT-A treatment may be gradually discontinued, while being ready to start it again if relapses occur. This tendency was more evident during the third year from the commencement of OBT-A, when only 10% of the clinicians maintained the 3-month interval between treatment cycles, nearly 20% of them extended the interval duration to 4-6 months, while the majority relied on the pattern of headache reported in the patients’ headache diaries to make decisions about treatment timing. Schedule variability after the first year of prophylaxis with OBT-A likely reflects the clinicians’ intention to comply with current recommendations, according to which OBT-A treatment should be interrupted when CM reverts to an episodic pattern (i.e., < 15 days with headache/month) [11, 19] and, at the same time, the fear that treatment discontinuation might revert episodic migraine to chronicity.

Identification of the best timing for treatment discontinuation is crucial for the therapeutic program with OBT-A. The PREEMPT study showed a progressive increase in the efficacy of OBT-A over time [15], but the relatively short treatment duration (14 months) of the trial did not provide data about long-term outcomes. In a recent real-life study, Guerzoni and coworkers have shown that the benefits in terms of headache days and QoL progressively increased during at least 7 cycles (18 months) of treatment with OBT-A [31]. Progressively increasing benefits for up to 24 months in terms of reduced number of headache days and decreased use of acute medications have been reported also by Negro and coworkers [32]. The limited evidence currently available suggests that treatment discontinuation performed once the frequency of headache days is <15 days/month, as currently recommended [19], results in worsening of headache, decrease in QoL [31] and the need to reinitiate treatment [47]. The decision to discontinue treatment has a heavy burden of consequences, given that CM can be highly refractory to treatment, disabling, and associated with comorbidities including depression, anxiety, and medication overuse [6, 7].

The need for a precise definition of response to treatment is another critical issue emerging from the survey. According to current guidelines for clinical trials in migraine [48], response to treatment for migraine is defined by a reduction ≥50% in the number of headache days. However a reduction ≥30% can also be clinically meaningful for CM [49]. Other authors have suggested different scores. Khalil and coworkers, for example, define as a responder any patient with either a 50% reduction in headache or migraine days or an increment in crystal clear days twice that of the baseline in a 30-day period (Hull criteria) [25]. According to our survey, 25% of the clinicians considered as a clinically meaningful response a ≥ 30% reduction in the number of the headache days. Furthermore, a smaller, but not irrelevant, percentage of clinicians (15.9%) felt that also a smaller reduction in the number of headache days (< 30%) might be worth considering when evaluating clinical response, provided that such reduction is associated with at least another indicator of improvement, including patient satisfaction with treatment, improvement of QoL, reduction in pain intensity, decrease in the number of medications used to relieve symptoms, or reduced duration of headache attacks. These findings, along with data from the literature [50], suggest that multiple efficacy measures should be considered to fully reflect clinical relevance, not only in regulatory clinical trials but also, and more importantly, in the real-life setting.

With regard to the definition of non-responders, the survey shows good agreement with the definition emerging from the literature (i.e., failure of at least 3 cycles of treatment) [50]. This finding also suggests that clinicians are aware of the putative mechanism of action of OBT-A, which is likely to require a prolonged time interval to revert the changes associated with the progression of migraine to chronicity [51].

Not surprisingly, the survey indicates that the treatment with OBT-A is usually initiated after the failure of several types of prophylactic agents (> 3 agents, according to 60% of clinicians), as current guidelines recommend OBT-A only for patients who have failed to respond to, or have not tolerated at least 3 prior pharmacologic prophylactic therapies [19]. However, according to our findings, most clinicians claimed, based on their experience, that the efficacy of OBT-A seems greater when the treatment is administered earlier in the course of CM. This impression is in agreement with previously published data. The pooled analysis of the PREEMPT 1 and 2 trials, for example, showed an increased benefit in patients who initiated OBT-A earlier compared to those who were treated 6 months later [16]. Furthermore, Castrillo and colleagues, in a real-life study, reported a negative correlation between the reduction in pain intensity and the number of drug treatments received before initiating OBT-A [52]. Along with the data from the literature, our findings indicate that further effort is required to define whether the early administration of OBT-A is associated with increased efficacy in CM. Currently we can only speculate about the reasons why early treatment may be more beneficial. It is generally accepted that recurring migraine attacks induce peripheral and central sensitization [53]. In CM, sensitization phenomena are associated with progressive and more pervasive functional and neuroanatomical changes [54]. Early treatment with OBT-A, via the direct inhibition of peripheral neurotransmitter and neuropeptide release, and the possible interaction with the surface expression of relevant membrane receptors, may counteract these changes when they are only partially expressed thus preventing their consolidation [54]. At the same time, we cannot exclude that early-stage CM may be more likely to undergo spontaneous fluctuations and/or to improve spontaneously than long-term CM. The potential benefits of early administration are relevant, not only to alleviate migraine pain, but also to prevent the loss of productivity and the increased use of healthcare resources typically associated with CM [55, 56]. Appropriate studies are required to establish whether OBT-A should be a first-line option in the management of patients with CM as 47.6% of the clinicians in the present survey recommend.

The cost-effectiveness of OBT-A in CM has yet to be defined in ad hoc prospective, long-term studies. Evidence suggests that the use of OBT-A may be associated with a decrease in resource use. An observational study in 35 patients initiating prophylaxis with OBT-A found that the new treatment was associated with a reduction in visits to the emergency department by 87% [57]. A retrospective study based on a health care claims database has shown that OBT-A treatment is associated with a significantly lower likelihood of headache-related visits to the emergency department and of hospitalizations [58]. Furthermore, analysis of data from the PREEMPT program, using a Markov model applied to the UK health care system, led to the conclusion that OBT-A treatment in CM represents a cost-effective use of resources [59].

Notably, the present survey reported a better tolerability profile and a more favorable efficacy/safety ratio of OBT-A compared with other prophylactic agents for migraine. The favorable tolerability/safety profile of OBT-A has been supported by comparative trials with oral drugs for the prophylactic treatment of CM [60, 61], and by its extended use in clinical practice in other indications [62].

The present study has a number of limitations. The response rate to our survey (65%), although in line with the mean response rates of physicians to mailed questionnaires reported in the literature, was slightly below the threshold of 70% generally considered desirable for ensuring survey validity [37]. Despite these inherent limitations, we believe that the selection of survey participants, based on the proven expertise in the management of headache disorders, ensured that the source of information was reliable and qualified. Of note, most survey participants had several years of experience in the use of OBT-A for CM; in some cases, the duration of OBT-A use extended beyond the approval date (2013) of OBT-A in Italy, probably because of the off-label use of the drug or the participation in RCTs [63]. Our findings, which describe the status of OBT-A use in Italy, may not be applicable to the clinical practice in other countries. However, most items in the questionnaire addressed unresolved issues of general interest in the field of CM, and were articulated in a way that avoided the constraints of national regulations. We believe that the information produced by our survey may be useful for the design of appropriate studies in the near future.

## Conclusions

At three years from the approval of OBT-A for the prophylaxis of CM in Italy, this novel therapeutic option appears to be used in accordance with current recommendations. The majority of clinicians in the survey considered the efficacy/safety profile of OBT-A more favorable than that of oral prophylactic agents. Satisfaction with this approach was high among clinicians and, according to almost 50% of them, OBT-A should be offered as first-line treatment. Additional effort from the headache community is required to define response to treatment and to establish the optimal duration of prophylaxis with OBT-A. Such effort is crucial to further improve the therapeutic approach to CM.

## Abbreviations

ANIRCEF: Associazione Italiana per la Ricerca sulle Cefalee
EMA: European Medicines Agency
FDA: US Food and Drug Administration
HR-QoL: Health-related quality of life
OBT-A: Onabotulinumtoxin A
PREEMPT: Phase III Research Evaluating Migraine Prophylaxis Therapy-1
QoL: Quality of life
RCTs: Randomized clinical trials
SISC: Società Italiana per lo Studio delle Cefalee
